# The glucosaminidase domain of Atl – the major *Staphylococcus aureus* autolysin – has DNA-binding activity

**DOI:** 10.1002/mbo3.165

**Published:** 2014-03-03

**Authors:** Inês R Grilo, Ana Madalena Ludovice, Alexander Tomasz, Hermínia de Lencastre, Rita G Sobral

**Affiliations:** 1 Laboratory of Molecular Genetics, Instituto de Tecnologia Química e Biológica da Universidade Nova de Lisboa2780, Oeiras, Portugal; 2 Departamento de Ciências da Vida, Faculdade de Ciências e Tecnologia, Universidade Nova de LisboaQuinta da Torre, 2829-516, Caparica, Portugal; 3 Laboratory of Microbiology and Infectious Diseases, The Rockefeller University1230 York Avenue, New York, New York, 10065; 4 Centro de Recursos Microbiológicos (CREM), Departamento de Ciências da Vida, Faculdade de Ciências e Tecnologia, Universidade Nova de LisboaQuinta da Torre, 2829-516, Caparica, Portugal

**Keywords:** Atl autolysin, DNA binding, protein-DNA interaction, *Staphylococcus aureus*

## Abstract

In this communication, we describe evidence demonstrating the capacity of Atl, the major *Staphylococcus aureus* autolytic enzyme to bind DNA. Electrophoretic mobility shift assays (EMSA) show that both the Atl protein and the endo-*β*-N-acetylglucosaminidase (GL) domain were able to bind DNA of nonspecific sequence. The implications of this unexpected observation for the physiology of *S. aureus* remain to be explored.

## Introduction

In *Staphylococcus aureus*, the major peptidoglycan hydrolase is Atl, a bifunctional protein (Oshida et al. 1995) with amidase (AM) and endo-*β*-*N*-acetylglucosaminidase (GL) domains, not only primarily involved in the separation of daughter cells during cell division but also contributing to cell wall turnover and antibiotic-induced lysis (Foster 1995; Sugai et al. 1995, 1997). The *atl* gene is translated as a single propeptide, which is secreted and proteolytically processed extracellularly, yielding the mature forms of AM (∼63 kDa) and GL (∼51 kDa). Besides the two catalytic domains, the Atl protein also harbors a N-terminal signal peptide and three repeat regions (R_1_, R_2_, and R_3_) located between the two catalytic domains (Oshida et al. 1995). After secretion, the thus far uncharacterized extracellular processing yields the mature AM associated to repeats R_1_ and R_2_, and GL associated to R_3_. Although these repeat regions show no lytic activity, they are responsible for attachment and substrate recognition (Biswas et al. 2006; Zoll et al. 2012).

The binding of both AM and GL to the staphylococcal surface occurs at precise locations of the equatorial surface rings, not only at the septum of dividing cells but also at a perpendicular surface ring that marks the future cell division site (Sugai et al. 1997; Baba and Schneewind 1998). Wall teichoic acids prevent the binding of Atl-derived proteins, targeting them to the septal region through an avoidance strategy (Schlag et al. 2010).

Most data available in the literature are related to the characterization of the AM domain of this enzyme, which catalyzes the cleavage of the amide bond between N-acetylmuramic acid and L-alanine residues of the stem peptide (Biswas et al. 2006). The structure resolution of AmiE of *Staphylococcus epidermidis* AtlE was a major contribution to the elucidation of the mechanisms of peptidoglycan attachment and catalysis by AM (Zoll et al. 2010). In contrast, only few reports are available on the activity and role of the GL domain, which is involved in the cleavage of the glycosidic bonds between the sugar molecules – N-acetylmuramic acid and N-acetylglucosamine – in the peptidoglycan backbone.

As expected from the multiple physiological roles of Atl, *S. aureus* mutants in the *atl* gene exhibit a variety of phenotypic alterations that include a disordered division pattern; formation of large cell clusters indicating defective cell separation; a rough outer surface; production of lower amounts of secreted and cell wall-bound proteins, and impairment of biofilm formation (Oshida and Tomasz 1992; Oshida et al. 1995; Sugai et al. 1997; Biswas et al. 2006).

In a recent series of exploratory experiments, we made the unexpected observation that the Atl protein – more specifically its GL domain – can bind DNA of unspecific sequence. The DNA-binding capacity of a peptidoglycan hydrolytic enzyme has not been previously reported, and the physiological implications of such interaction could be numerous and remain to be explored.

## Experimental Procedures

### Bacterial strains, plasmids, and growth conditions

Bacterial strains and plasmids used in this study are listed in Table 1. All strains were grown at 37°C with aeration, in the following media: tryptic soy broth (TSB) or tryptic soy agar (TSA) (Difco Laboratories, Detroit, MI) for *S. aureus* strains, Lysogeny broth (LB) or Lysogeny agar (LA) (Difco Laboratories) for *Escherichia coli* strains, and Brain Heart Infusion broth (BHI) or BHI-agar (Difco Laboratories) for *Micrococcus luteus*.

**Table 1:** Strains and plasmids used in this study.

Strain or plasmid	Description	Source or reference
*Staphylococcus aureus*		
RN4220	Mc^s^; restriction negative	Novick (1991)
COL	Homogenous Mc^r^ (MIC, 1600 *μ*g mL^−1^); Em^s^	Rockefeller University Collection
*Escherichia coli*		
DH5*α*	*recA endA1 gyrA96 thi-1 hsdR17 supE44 relA1* ϕ80 Δ*lacZ*ΔM15	Invitrogen
BL21(DE3)	*F*^*–*^*ompT gal dcm lon hsdS*_*B*_(*r*_*B*_^*−*^*m*_*B*_^*−*^) *λ*(*DE3* [*lacI lacUV5-T7 gene 1 ind1 sam7 nin5*])	Invitrogen
Plasmids		
pET28a(+)	*E. coli* expression vector	Novagen
pET-AMR_1–3_GL	pET28a(+) expressing AMR_1-3_GL as a N-terminal His-tag fusion (fragment amplified with Pexp1 and Pexp4)	This study
pET-AMR_1–2_	pET28a(+) expressing AMR_1-2_ protein as a N-terminal His-tag fusion. pET-AMR_1-3_GL was used to insert a stop codon by directed mutagenesis with primers Pexp_stop1 and Pexp_stop2	This study
pET-AM	pET28a(+) expressing AM as a N-terminal His-tag fusion (fragment amplified with Pexp1 and Pexp5)	This study
pET-R_3_GL	pET28a(+) expressing R_3_GL as a N-terminal His-tag fusion (fragment amplified with Pexp2 and Pexp4)	This study
pET-GL	pET28a(+) expressing GL as a N-terminal His-tag fusion (fragment amplified with Pexp3 and Pexp4)	This study
pET-GL_N_	pET28a(+) expressing GL_N_ as a N-terminal His-tag fusion (fragment amplified with Pexp3 and Pexp7)	This study
pET-GL_C_	pET28a(+) expressing GL_C_ as a N-terminal His-tag fusion (fragment amplified with Pexp6 and Pexp4)	This study

Antibiotics erythromycin (10 *μ*g mL^−1^), ampicillin (100 *μ*g mL^−1^) and kanamycin (30 *μ*g mL^−1^) were used when needed as recommended by the manufacturer (Sigma-Aldrich, St. Louis, MO).

### DNA methods

Restriction enzymes (New England Biolabs, Beverly, MA) were used as recommended by the manufacturer. Routine PCR (polymerase chain reaction) amplification was performed with GoTaq Flexi DNA polymerase (Promega, Madison, WI) and PCR amplification for cloning purposes was performed using Phusion High Fidelity DNA polymerase (Finnzymes, Vantaa, Finland).

For plasmid DNA extraction, High pure Plasmid Purification Kit (Roche, Basel, Switzerland) was used. PCR and digestion products were purified with High pure PCR Purification Kit (Roche). Ligation reactions were performed with Rapid DNA Ligation kit (Roche).

### DNA-binding protein purification assays

Cells were harvested at midexponential phase and lysed as described (Fournier et al. 2000). The lysate was dialyzed using 3500 MWCO slide-a-lyzer cassettes (Pierce Biotechnology, Rockford, IL) against the dialysis buffer described.

The biotinylated DNA fragments were amplified using DNA from *S. aureus* strain COL (Table1) and primer pairs pmurFGS4 and Biotin-pddlAlow3 (Fragment A – 238 bp of the promoter of *ddlA-murF* operon), pFptaF and Biotin-pFptaR (Fragment B – 422 bp of the promoter of *pta* gene), and Biotin-ptaF and ptaR (Fragment C – 314 bp of an internal region of *pta* gene).

The biotinylated fragments were bound to streptavidin-coated magnetic Dynabeads (Invitrogen Dynal AS, Oslo, Norway) as recommended by the manufacturer. The beads were washed in B&W buffer and resuspended in 2× B&W buffer, with an equal volume of the biotinylated DNA. After incubation with constant rotation, the DNA-coated beads were washed and separated with a magnet (Invitrogen Dynal AS).

Binding reactions were performed as described (Ender et al. 2009), with minor adjustments: DNA-coated beads were incubated with 600 *μ*g of crude extracts in protein binding buffer at RT with rotation. Following several washes, the beads were boiled in 0.1% sodium dodecyl sulfate (SDS) and the eluted proteins were separated by SDS polyacrylamide gel electrophoresis (SDS-PAGE) and stained with Coomassie blue silver (Candiano et al. 2004). Protein bands were analyzed by mass spectrometry (MALDI-TOF/TOF) at the Mass Spectrometry Laboratory, Analytical Services Unit, ITQB, UNL.

To assess binding specificity, salmon sperm DNA (Sigma-Aldrich) was added to the extracts (200-fold molar excess) prior to incubation with the beads.

### Construction of recombinant plasmids for protein expression

Different *atl* DNA fragments were amplified by PCR using DNA from *S. aureus* strain COL as template and specific primers (Table S1), which included the appropriate restriction sites for cloning. The fragments were purified, digested with respective restriction enzymes, and cloned into pET28a (+) plasmid (Novagen, Merck Millipore, Nottingham, UK), using *E. coli* DH5*α* in-house prepared competent cells. The correct recombinant plasmids (Table 1) were confirmed by restriction analysis and sequencing, and used to transform in-house prepared *E. coli* BL21(DE3) competent cells for protein expression.

To construct the His6-AMR_1_R_2_ recombinant protein, plasmid pET28a-AMR_1_R_2_R_3_GL was used as template for site-directed mutagenesis using primers Pexp_stop1 and Pexp_stop2 (Table S1), to change codon 175 from AAA (Lys) to TAA (stop codon).

### Protein expression and purification

The constructed proteins were expressed as N-terminal 6xHis-tag fusion proteins. Expression was performed using the auto-induction expression method (Studier 2005). Cells were harvested at late-log phase and resuspended in 1/10 volume of Purification Lysis buffer containing 10 UmL^−1^ of benzonase nuclease (Novagen, Merck Millipore) and Complete-Mini Protease Inhibitor Cocktail EDTA-free Tablet (Roche). After cell disruption, the lysates were cleared. The recombinant proteins were all soluble and, therefore, present in the supernatant.

The recombinant proteins were purified using Ni-NTA agarose columns (Qiagen, Hilden, Germany) under native conditions, according to the manufacturer's instructions. The expression and purification yields were monitored by SDS-PAGE. The most concentrated elution fractions were dialyzed in a 3500 MWCO snakeskin dialysis tubing (Pierce Biotechnology) at 4°C, against 100 mmol/L Tris pH 7.5.

### Zymogram analysis

Heat-inactivated cells were prepared as described (Yokoi et al. 2008), with some modifications. *S. aureus* and *M. luteus* were grown to mid-exponential phase and cells were recovered by centrifugation, washed twice, resuspended in cold water, and heat inactivated by autoclaving at 121°C for 15 min. After cooling, the heat-inactivated cells were washed twice with cold water, and kept in 0.05% sodium azide.

Zymograms were performed in 12% SDS-PAGE gels with the incorporation of 2 × 10^9^ heat-inactivated cells of *S. aureus* or *M. luteus,* and 100 ng of each protein was used. After electrophoresis, the zymogram gels were washed thrice with water, and incubated with renaturation buffer (0.1% Triton X-100, 10 mmol/L CaCl_2_, 10 mmol/L MgCl_2_, 50 mmol/L Tris-HCl pH 7.5) for an hour. Gels were stained with methylene blue staining (1% methylene blue in 0.01% KOH) and destained with water.

### Electrophoretic mobility shift assays

Gel-shift assays were performed using purified recombinant proteins and a 5′-fluorescein-labeled DNA fragment A, amplified by PCR, using primers Fluo-pddlalow3 and pmurFGS4 (Table S1). Prior to the binding reaction, the DNA fragments were purified.

The binding reaction components were combined to a final volume of 20 *μ*L, in the following order: ultrapure water, 10× EMSA binding buffer (100 mmol/L Tris pH 7.5, 500 mmol/L KCl, 10 mmol/L DTT [Dithiothreitol]), 10 nmol/L of target DNA, purified recombinant proteins in increasing amounts, and 200-fold molar excess of unspecific DNA (low-molecular weight salmon sperm DNA, Sigma-Aldrich), when applicable. Final protein concentrations ranged from 0 to 1 *μ*mol/L. Following a 30-min incubation period at room temperature, the binding reactions were separated by electrophoresis in 1% agarose gels (1× TAE [Tris-acetate-EDTA buffer]). Visualization was performed using a Fuji Fluorescent Analyzer TLA-5100.

### Lysis assays of heat-inactivated cells

Lysis assays were performed in sterile nontreated 96-well microplates (Brand, Wertheim, Germany) at 37°C with shaking (400 rpm) for 2 h. Heat-inactivated cells of *S. aureus* strain COL and *M. luteus*, were prepared as above, and were resuspended in 50 mmol/L Tris pH 8.0 to an initial OD_600_ of 0.3. Low-molecular weight salmon sperm DNA and purified proteins AMR_1–2_ and R_3_GL were added to the wells at the beginning of the assay at different concentrations. Three biological replicates were performed in triplicate in an Infinite F200 PRO microplate reader (Tecan Group Ltd., Männedorf, Switzerland).

The decrease in OD_600_ observed for each protein/DNA concentrations was calculated as a percentage of the initial OD_600_ value.

### Statistical analysis

Lysis assays data were analyzed by performing a one-way analyses of variance (ANOVA) with Tukey multiple comparison tests, using GraphPad Prism (La Jolla, CA).

## Results and Discussion

### Identification of the DNA-binding activity of *S. aureus* autolysin Atl

A DNA-binding protein pull-down assay was performed using total crude cell extracts of strain COL and the biotinylated DNA fragment A immobilized to the surface of streptavidin-coated magnetic particles. The proteins that bound to the biotinylated DNA fragment were eluted and separated by SDS-PAGE.

**Figure 1:**
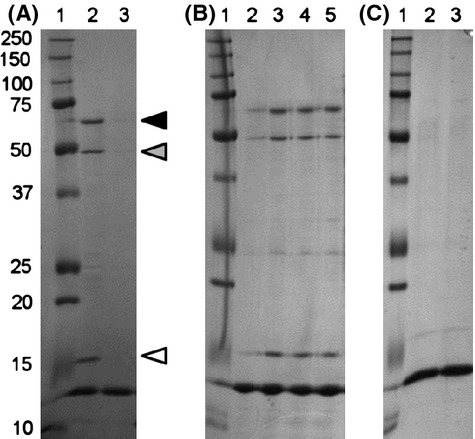
Identification of DNA-binding proteins from *Staphylococcus aureus*. Crude extracts from strain COL were incubated with DNA-coated magnetic beads. The beads-DNA-protein complexes were recovered using a magnet, and the proteins associated with the DNA were separated by SDS-PAGE. Lane 1: Protein marker, molecular mass in kDa. (A) Lane 2: beads coated with DNA fragment from *ddlA-murF* promoter. Lane 3: uncoated beads. Solid triangle – 70 kDa bands; gray triangle – 50 kDa band, GL domain; white triangle – 15 kDa band, IsaB protein. (B) Lane 2: uncoated beads; Lane 3: beads coated with DNA fragment from *ddlA-murF* promoter; Lane 4: beads coated with DNA fragment from *pta* gene; Lane 5: beads coated with DNA fragment from *pta* promoter. (C) Salmon sperm DNA was added to the protein extracts prior to incubation with the DNA-coated beads. Lane 2: beads coated with DNA fragment from *ddlA-murF* promoter. Lane 3: beads coated with DNA fragment from *pta* gene.

Three major bands were obtained with approximate molecular masses of 70, 50, and 15 kDa. The corresponding proteins bound to the DNA-coated beads but not to uncoated beads (Fig. 1A).

To test the DNA sequence binding-specificity of these proteins, the pull-down assays were repeated using other biotinylated DNA fragments as bait molecules: fragment B, encompassing the promoter of the housekeeping gene *pta*, and fragment C, enclosing an internal region of the *pta* gene. The band pattern was identical to the one previously obtained (Fig. 1B). As a control for specificity, the crude extracts were preincubated with salmon sperm DNA in excess, prior to incubation with the DNA-coated beads (Fig. 1C). Under these conditions, no protein was found to associate with the biotinylated DNA fragments. Together, these assays indicated that the proteins bound to double-stranded DNA but in a sequence-independent manner.

Bands of interest were excised from the gel for identification by mass spectrometry (Fig. 1A). The higher molecular mass band (70 kDa) was composed of three bands, which although distinct, had very similar molecular masses and, therefore, could not be independently analyzed. The 15 and 50 kDa bands were identified as IsaB, immunodominant antigen B, and the mannosyl-glycoprotein endo-β-N-acetylglucosaminidase domain (GL) of the bifunctional autolysin Atl, respectively.

The DNA-binding activity of IsaB, the 15 kDa protein, has been previously reported (Mackey-Lawrence et al. 2009). However, the Atl protein, the major autolysin responsible for the separation of daughter cells in *S. aureus*, had not been previously described to bind any type of nucleic acid.

### Assessment of DNA-binding activity by gel-shift analysis

**Figure 2:**
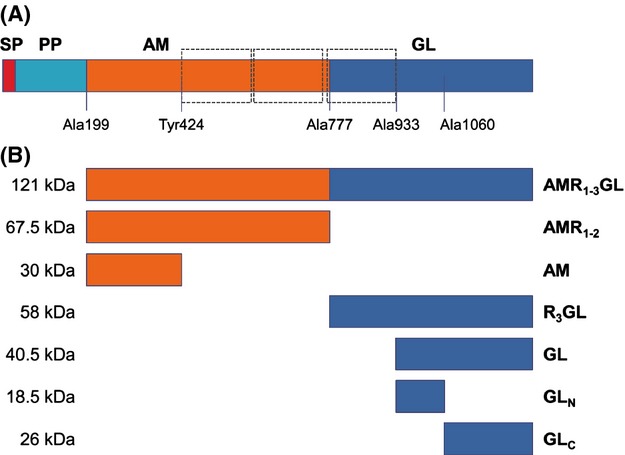
Structural organization of *Staphylococcus aureus* Atl and His-tagged recombinant proteins. (A) Atl is a bifunctional protein composed of a signal peptide (SP), a propeptide (PP), and the catalytic domains amidase (AM) and glucosaminidase (GL). Repeat regions are indicated in dashed boxes. The first amino acid residue of each recombinant protein is indicated, except for Tyr424, which corresponds to the last amino acid of AM protein. (B) Constructed recombinant proteins. Approximate molecular masses are indicated.

In total crude extracts, the GL domain was observed to bind to DNA in a sequence-unspecific manner. In order to determine if the DNA-binding activity was a characteristic intrinsic to the GL domain and not dependent on other factors present in the crude extract, gel-shift assays were performed with purified recombinant proteins. Two GL recombinant proteins were constructed and purified: R_3_GL and GL without the R_3_ repeat (Fig. 2). The repeat units have no lytic activity, but they are responsible for attachment to peptidoglycan (Biswas et al. 2006), to lipoteichoic acids (Zoll et al. 2012), fibronectin and vitronectin (Heilmann et al. 1997). Furthermore, each repeat region harbors a prokaryotic SH3-related domain, structurally similar to conserved eukaryotic SH3 domains, being the SH3 fold able to bind to DNA and RNA (Gao et al. 1998; Kishan and Agrawal 2005).

**Figure 3:**
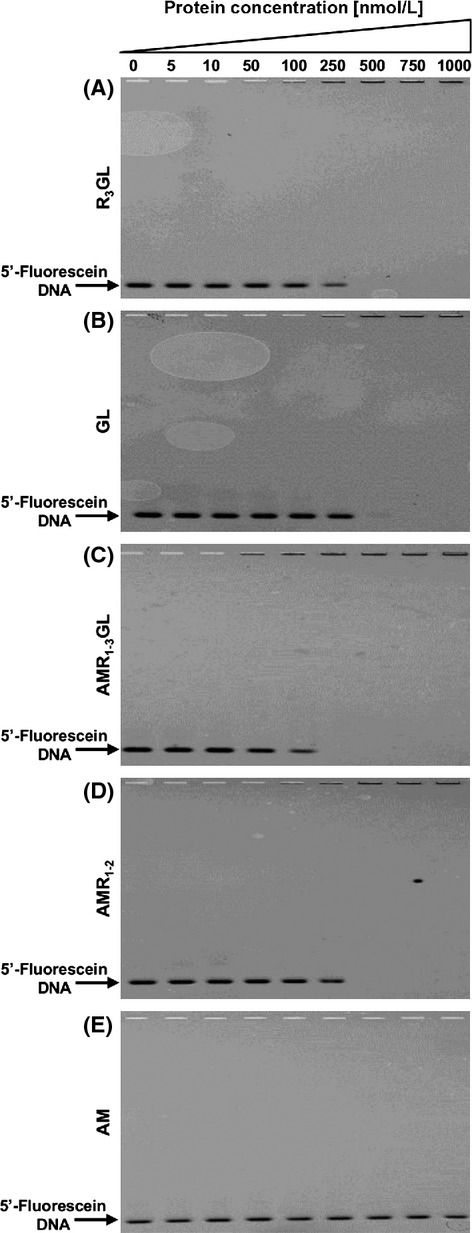
Electrophoretic mobility shift assays with recombinant proteins. 10 nmol/L of DNA 5′-fluorescein-labeled fragment A was mixed with increasing amounts of the different recombinant proteins, and the ability to retain DNA was visualized. (A) R_3_GL – GL domain; (B) GL – GL domain without the repeat region; (C) AMR_1–3_GL – entire Atl recombinant protein (lacking the SP and PP sequences); (D) AMR_1–2_ – AM domain with the repeat region; and (E) AM – AM domain without the repeats.

In order to determine if the DNA-binding activity observed was the result of the presence of the repeat region, EMSA were performed using the R_3_GL and GL recombinant proteins (Fig. 3A and B). Both proteins were able to completely shift 10 nmol/L of the DNA fragment A for a protein concentration of 500 nmol/L, indicating that the GL catalytic domain has DNA-binding activity independent of the presence of the R_3_ repeat unit.

### DNA binding to the complete Atl protein

As the Atl protein is processed extracellularly, the GL domain in its mature form should only be present at the outer surface of the cell or in the external environment. To address the question if preprocessed Atl was also able to bind DNA, attempts were made to obtain the complete His-tagged Atl protein without the signal peptide. The successful approach for this involved construction of the Atl protein without the signal peptide and the propeptide (AMR_1–3_GL). This recombinant protein, at a concentration of 250 nmol/L, proved capable of shifting 10 nmol/L of DNA fragment A (Fig. 3C). This result raises the possibility that the Atl protein may be able to bind to DNA intracellularly as well. Furthermore, in order to shift 10 nmol/L of DNA, 250 nmol/L of the whole Atl protein was sufficient while 500 nmol/L of GL was necessary.

### DNA binding to the AM domain of Atl

In order to determine if the AM domain also has DNA-binding activity that could contribute to the activity of the full protein, recombinant proteins for the AM domain including the R_1_R_2_ repeat regions (AMR_1–2_) and the AM domain without the R regions (AM) were constructed, expressed, and purified (Fig. 2). Although the AMR_1–2_ protein shifted DNA at a concentration of approximately 500 nmol/L, the corresponding recombinant protein without the repeats did not shift DNA at any of the concentrations and conditions tested (Fig. 3D and E, respectively). This observation suggests that the DNA-binding activity of the AM domain is, in contrast to the GL domain, restricted to the repeat regions.

### DNA binding to the catalytic domain of GL

**Figure 4:**
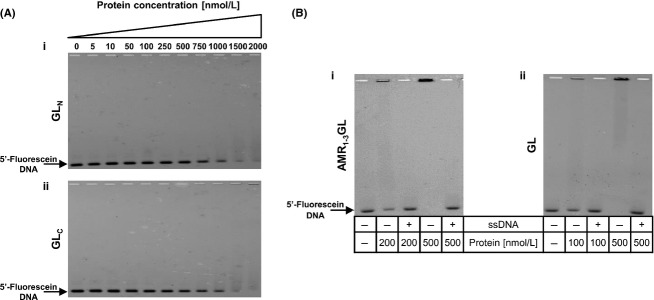
Electrophoretic mobility shift assays. (A) Performed with recombinant proteins corresponding to (i) the N-terminal – GL_N_ and (ii) C-terminal – GL_C_ regions of the catalytic domain of GL. Both proteins were able to shift 10 nmol/L of the 5′-fluorescein-labeled DNA fragment A, at high protein concentrations. (B) Competition electrophoretic mobility shift assays. All binding reactions were performed with 10 nmol L^−1^ of 5′-fluorescein-labeled fragment A, and ∼200-fold molar excess of unspecific DNA (low-molecular weight salmon sperm DNA, ssDNA) was added in the lanes specified. (i) AMR_1–3_GL protein, (ii) GL protein.

To determine if the catalytic domain of GL was involved with the DNA-binding activity, two recombinant proteins were constructed encompassing the first 127 amino acids immediately after the R_3_ repeat (GL_N_) and the following 197 amino acids of the C-terminus of Atl (GL_C_) (Fig. 2). In the first reference to the Atl protein by Oshida and Tomasz (1992), the glucosaminidase lytic activity was found to be located in a GL C-terminal fragment encoded by the DNA sequence downstream from a SphI restriction site. This restriction site overlays the position Ala1060 (Oshida et al. 1995). Accordingly, GL_N_ and GL_C_ proteins correspond to the N-terminal and C-terminal regions of the catalytic domain of GL relative to the amino acid Ala1060. However, the EMSA results showed that both proteins were able to bind to DNA albeit at much higher protein concentrations (Fig. 4A), indicating that both protein regions are necessary for the full DNA-binding activity.

### Lack of sequence specificity in DNA binding

For all proteins which presented a retardation of the DNA, addition of 200-fold molar excess of nonspecific competitor DNA to the reaction mixture abolished the shift (representative gel shifts are shown in Fig. 4B), indicating that the interaction is not specific for DNA sequence. Furthermore, DNA fragments B and C, of unrelated sequence were also tested in EMSA and produced the same results (data not shown).

These findings indicate that the DNA-binding activity of Atl protein is independent of the DNA sequence and due primarily to the GL catalytic domain and to the repeat regions.

### Lytic activity of the recombinant proteins

**Figure 5:**
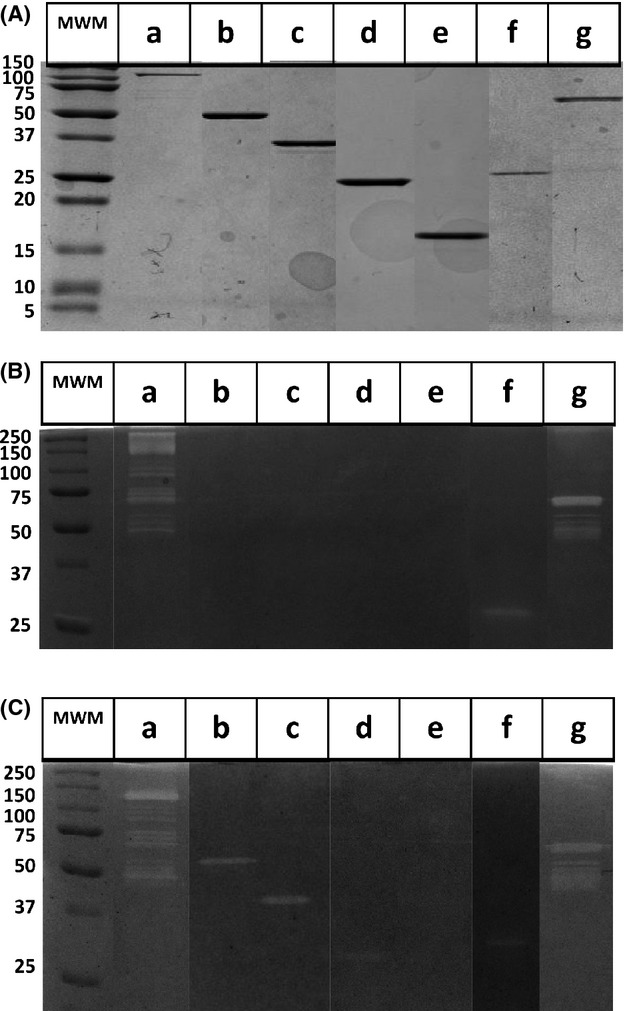
Purified recombinant proteins and assessment of hydrolytic activity. (A) Staining of the purified recombinant proteins with Coomassie Brilliant Blue after SDS-PAGE and the corresponding zymograms performed with incorporated heat-inactivated (B) *Staphylococcus aureus* cells and (C) *Micrococcus luteus* cells. Proteins: (a) AMR_1–3_GL, (b) R_3_GL, (c) GL, (d) GL_C_, (e) GL_N_, (f) AM, (g) AMR_1–2_.

The recombinant proteins retained their native cell wall hydrolytic activity, as shown by zymograms performed with heat-inactivated cells of *S. aureus* or of *M. luteus* (Fig. 5B and C, respectively). The clear zones observed in the zymograms corresponded to the band sizes determined by Coomassie blue staining (Fig. 5A). As expected, AMR_1–3_GL, AMR_1–2_, and AM showed hydrolytic activity against heat-inactivated cells of both *S. aureus* and *M. luteus* while R_3_GL, GL and GL_C_ were only capable to lyse *M. luteus* (Oshida et al. 1995). Protein GL_N_ that did not include the lytic domain had no hydrolytic capacity.

The activity of the recombinant proteins was further confirmed in vivo*,* demonstrating their capacity to catalyze separation of cell clusters and restoring the parental phenotype (M. L. Atilano, P. M. Pereira, F. Vaz, M. J. Catalão, P. Reed, I. R. Grilo, R. G. Sobral, P. Ligoxygakis, M. G. Pinho and S. R. Filipe, unpublished data).

### GL-DNA-binding activity does not affect GL lytic capacity

**Figure 6:**
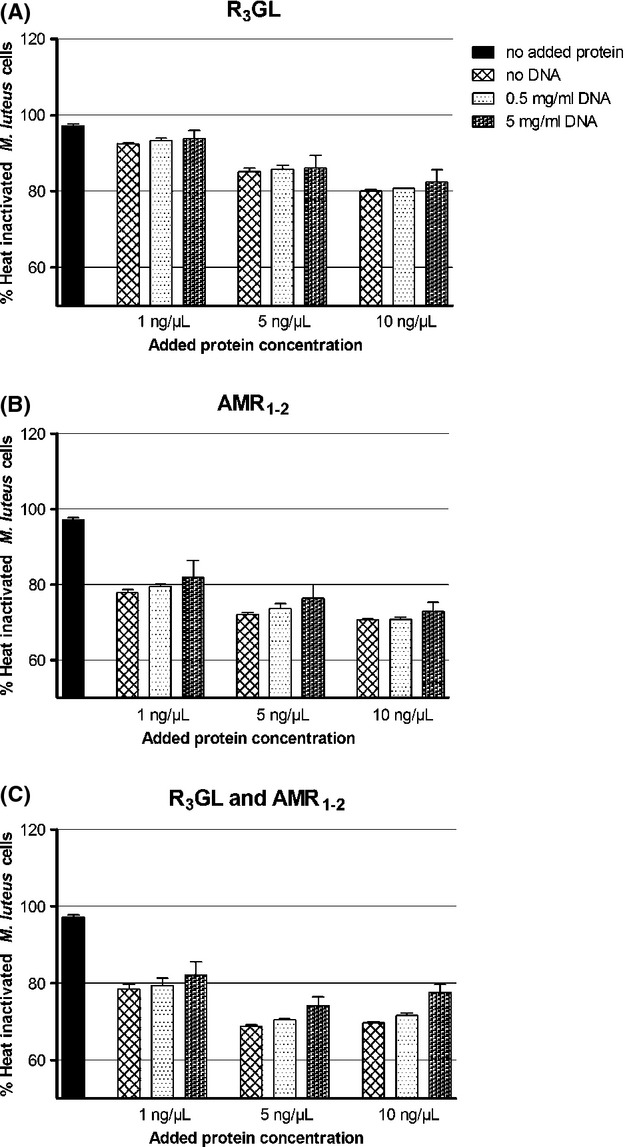
Lysis assays of heat-inactivated cells. Recombinant proteins were added at different concentrations to heat-inactivated *Micrococcus luteus* cells and lysis was followed by measuring the decrease in OD_600_. Different concentrations of added DNA did not affect significantly the lysis of cells. (A) Protein R_3_GL. (B) Protein AMR_1–2_. (C) Proteins R_3_GL and AMR_1–2_.

The primary physiological role of Atl is its hydrolytic activity on the staphylococcal peptidoglycan. To determine if the DNA-binding capacity of the Atl domains influences their lytic activity, we performed lytic assays using heat-inactivated cells of *M. luteus* as substrate in combination with different DNA concentrations and the AMR_1–2_ and R_3_GL proteins as catalysts of cell wall degradation (Fig. 6). No significant effect was observed for any of the DNA concentrations tested, and the same result was obtained with heat-inactivated cells of *S. aureus* (data not shown).

## Discussion

The major autolysin of *S. aureus* – Atl *–* plays a variety of roles in the physiology of this bacterium: deletion mutants of *atl* form large cell clusters; have a “rough” outer surface; produce lower amounts of secreted and cell wall-bound proteins; and are impaired in biofilm formation (Oshida and Tomasz 1992; Oshida et al. 1995; Sugai et al. 1997; Biswas et al. 2006). The novel observations described in this communication show that Atl, specifically its GL domain, also plays an additional role in binding DNA.

Atl exists intracellularly as an unprocessed protein but after secretion it becomes attached to the external cell surface and undergoes proteolytic cleavage into two smaller polypeptides, which have hydrolytic activity. The observations described in this communication indicate that both forms of the hydrolase – the processed and the unprocessed – were able to bind DNA molecules, suggesting multiple possible roles for such activity.

EMSA showed that the DNA-binding activity was not sequence specific and did not require interaction with other proteins. DNA-binding activity was present in the unprocessed Atl protein, in the catalytic region of the GL domain and in the repeats as well. While the repeats are known to be able to attach to a wide variety of different molecules, this activity could only account for part of the total DNA-binding described in this report. The GL catalytic domain showed DNA-binding activity independent of the presence of the R_3_ repeat unit. In contrast, DNA-binding activity of the AM domain appears to be restricted to the repeats, and DNA binding to the catalytic domain was not observed.

In the context of *S. aureus* cell division one might speculate that the binding of the whole Atl protein to DNA molecules may be involved in the process of anchoring the chromosome to the cell envelope in order to orient it during cell division (Toro and Shapiro 2010). If this were the case, one would expect to detect in Atl mutants cell division abnormalities such as asymmetric septa and slower division rate. However, tests with the Atl mutant RUSAL9 (Oshida and Tomasz 1992) detected no abnormalities (Sugai et al. 1997). Therefore, we favor the hypothesis that the DNA-binding capacity of Atl described in this report is primarily related to the capture of extracellular DNA molecules. The possibility that the DNA binding of GL described here may be involved with the cellular uptake of DNA is presently being explored. DNA-mediated genetic transformation was recently reported in *S. aureus* (Morikawa et al. 2012).

Furthermore, *S. aureus atl* mutants are impaired in biofilm formation (Biswas et al. 2006); GL and AM are both able to independently and partially restore this phenotype although the full complementation requires both domains (Bose et al. 2012). The GL-DNA interaction described in this report may play a role in the formation and/or stability of biofilms.

The main biological role of this association remains to be explored, and further experiments are in progress to test the effect of this interaction in biofilm formation and in cellular DNA uptake.

## Supporting Information

Table S1 Oligonucleotide primers used in this study.
