# Supplementary material for: The glucosaminidase domain of Atl – the major Staphylococcus aureus autolysin – has DNA-binding activity
Source: Microbiologyopen. 2014 Mar 3;3(2):247–56. doi: 10.1002/mbo3.165 (PMC3996572; doi:10.1002/mbo3.165)
Supplement: Table S1 — Oligonucleotide primers used in this study. [file mbo30003-0247-sd1.doc]

**SUPPORTING INFORMATION**

Table S1 – Oligonucleotide primers used in this study.

| Primer | Nucleotide Sequence (5’ – 3’) | Source |
| --- | --- | --- |
| *Recombinant protein expression* | | |
| Pexp1 | CCAGGATCCGCTTCAGCACAACCAAGATCAG | This study |
| Pexp2 | CGTGGATCCGCTTATACTGTTACTAAACC | This study |
| Pexp3 | CGTGGATCCGCACCAACTGCTGTGAAACC | This study |
| Pexp4 | CCAGTCGACTTATTTATATTGTGGGATGTCG | This study |
| Pexp5 | CCAGTCGACTTAGGTAGTTGTAGATTGCG | This study |
| Pexp6 | CCAGGATCCGCAATGGATACGAAGCGTTTAGC | This study |
| Pexp7 | CCTGTCGACTTAATGCTTAACATCATTAAAGTTAGC | This study |
| Pexp_stop1 | GCAGTAGCACAACCAAAAACAGCTGTAtaaGCTTATACTGTTACTAAACCACAAACG | This study |
| Pexp_stop2 | CGTTTGTGGTTTAGTAACAGTATAAGCttaTACAGCTGTTTTTGGTTGTGCTACTGC | This study |
| *Electrophoretic Mobility Shift Assays and DNA Binding protein Purification Assays* | | |
| PmurFGS4 | CACAGTGATATCAGCTATAG | This study |
| PmurFGS4-comp | CTATAGCTGATATCACTGTG | This study |
| pmurFGS2 | CAATTAACAATAGGACTAAATC | This study |
| Biotin-PddlAlow3 | biot-CCTCCAATGATATATCAGGG | This study |
| PFptaF | CCCAAGCTTGATCACCAGATTTTG | This study |
| Biotin-PFptaR | biot-CGTTCGTCCTCTCCTTCAGG | This study |
| Biotin-ptaF | biot-CCTGAAGGAGAGGACGAACG | This study |
| ptaR | GCTGCACCACTAACTAAACC | This study |
| PddlAlow3 | CCTCCAATGATATATCAGGG | This study |
| Fluorescein-PddlAlow3 | fluo-CCTCCAATGATATATCAGGG | This study |

The restriction sequences included in the primers are underlined.
